# Mt10-CVB3 Vaccine Virus Protects against CVB4 Infection by Inducing Cross-Reactive, Antigen-Specific Immune Responses

**DOI:** 10.3390/microorganisms9112323

**Published:** 2021-11-10

**Authors:** Ninaad Lasrado, Rajkumar Arumugam, Mahima T. Rasquinha, Meghna Sur, David Steffen, Jay Reddy

**Affiliations:** School of Veterinary Medicine and Biomedical Sciences, University of Nebraska-Lincoln, Lincoln, NE 68503, USA; ninaad@huskers.unl.edu (N.L.); arrajkumar88@gmail.com (R.A.); mrasquinha2@huskers.unl.edu (M.T.R.); msur2@huskers.unl.edu (M.S.); dsteffen1@unl.edu (D.S.)

**Keywords:** vaccine, coxsackievirus B3, coxsackievirus B1, coxsackievirus B4, pancreatitis, cross protection

## Abstract

Group B coxsackieviruses (CVB) containing six serotypes, B1–B6, affect various organs, and multiple serotypes can induce similar diseases such as myocarditis and pancreatitis. Yet, no vaccines are currently available to prevent these infections. Translationally, the derivation of vaccines that offer protection against multiple serotypes is highly desired. In that direction, we recently reported the generation of an attenuated strain of CVB3, termed Mt10, which completely protects against both myocarditis and pancreatitis induced by the homologous wild-type CVB3 strain. Here, we report that the Mt10 vaccine can induce cross-protection against multiple CVB serotypes as demonstrated with CVB4. We note that the Mt10 vaccine could induce cross-reactive neutralizing antibodies (nABs) against both CVB1 and CVB4. In challenge studies with CVB4, the efficacy of the Mt10 vaccine was found to be 92%, as determined by histological evaluation of the heart and pancreas. Antibody responses induced in Mt10/CVB4 challenged animals indicated the persistence of cross-reactive nABs against CVB1, CVB3, and CVB4. Evaluation of antigen-specific immune responses revealed viral protein 1 (VP1)-reactive antibodies, predominantly IgG2a, IgG2b, IgG3, and IgG1. Similarly, by using major histocompatibility complex class II tetramers, we noted induction of VP1-specific CD4 T cells capable of producing multiple T cell cytokines, with interferon-γ being predominant. Finally, none of the vaccine recipients challenged with CVB4 revealed the presence of viral nucleic acid in the heart or pancreas. Taken together, our data suggest that the Mt10 vaccine can prevent infections caused by multiple CVB serotypes, paving the way for the development of monovalent CVB vaccines to prevent heart and pancreatic diseases of enteroviral origin.

## 1. Introduction

Coxsackieviruses belonging to the *Picornaviridae* family, and the genus Enterovirus are small (30 nm) non-enveloped, positive-sense, single-stranded RNA viruses. The genus Enterovirus includes group A and group B coxsackieviruses (CVB) that contain 23 and 6 serotypes, respectively, that affect various organs, with the potential for serotypes of both groups to cause neurological, cutaneous/mucosal, respiratory tract, and muscular diseases [1,2,3]. However, only CVBs are known to induce heart, pancreatic, and gastrointestinal diseases [1,4,5]. Importantly, CVB3 is mainly implicated in the causation of myocarditis that can lead to dilated cardiomyopathy (DCM) [6,7,8]. Likewise, although multiple serotypes (CVB1, CVB3, and CVB4) can induce pancreatitis, CVB1 and CVB4 infections may trigger type I diabetes (T1D) as demonstrated in the non-obese diabetic (NOD) mouse model and humans [9,10,11,12,13]. Despite the negative impact of CVB infections, vaccines are not available to prevent them, in part because their disease outcomes are not as devastating as those noted with some newly emerged viruses, such as Ebola, Zika, and severe acute respiratory syndrome coronavirus-2 (SARS-CoV-2) [14,15,16]. Nonetheless, the development of vaccines for infections like CVB is of paramount importance, especially for serotypes whose infections can lead to long-term chronic diseases such as DCM and T1D, as described above. Conversely, derivation of vaccines for each serotype is not feasible; rather, the development of monovalent vaccines that can prevent multiple serotypes may have more merit.

We recently reported the creation of an attenuated strain of CVB3, termed Mt10, that offered complete protection against both myocarditis and pancreatitis in A/J mice, which are highly susceptible to both diseases [17,18]. Essentially, the Mt10 vaccine, hereafter termed *vaccine virus*, was generated by introducing an H790A mutation within the viral protein 1 (VP1) region of the CVB3 viral canyon, where the coxsackievirus–adenovirus receptor (CAR) is expected to interact with the virus [17,19]. Based on this success, we sought to determine whether the vaccine virus can also induce cross-reactive immune responses and can protect against infection caused by other serotypes of CVB. Here, we report that the vaccine virus, in addition to inducing cross-reactive neutralizing antibody (nAB) responses to both CVB1 and CVB4, protects against CVB4 infection in A/J mice. The vaccine recipients challenged with CVB4 continue to show the persistence of cross-reactive nABs for the serotypes described above, with T-cell responses producing multiple T cell cytokines skewing towards interferon (IFN)-γ-producing T helper (Th)1 response.

## 2. Materials and Methods

### 2.1. Mice

Six-to-eight-week-old male and female A/J mice (H-2^a^) were procured from the Jackson Laboratory (Bar Harbor, ME, USA), maintained according to the institutional guidelines of the University of Nebraska-Lincoln, Lincoln, NE, and approved for animal studies by the university’s Institutional Animal Care and Use Committee. Infection studies were performed following biosafety level 2 guidelines, and euthanasia was performed using a carbon dioxide chamber as recommended by the Panel on Euthanasia of the American Veterinary Medical Association.

### 2.2. Peptides and Proteins

Peptides used in this study (VP1 681-700, RFDLELTFVITSTQQPSTTQ; VP1 721-740, PDKVDSYVWQTST NPSVFWT; RNase 43-56, VNTFVHESLADVQA) were synthesized by 9-fluorenylmethyloxycarbonyl chemistry (Neopeptide, Cambridge, MA, USA). Peptide purity was ascertained via high-performance liquid chromatography to be more than 90%, and peptide identity was confirmed by mass spectroscopy. Peptides were dissolved in ultra-pure water, and multiple aliquots were stored at −20 °C until further use. Full-length CVB3 VP1 (GenScript, Piscataway, NJ, USA) and keyhole limpet hemocyanin (KLH) protein (Sigma-Aldrich, St. Louis, MO, USA) were procured commercially.

### 2.3. Virus Propagation and Titration

LLC-MK2 cells or Vero cells (American type cell culture [ATCC], Manassas, VA, USA) were grown to 80–90% confluency. LLC-MK2 cells were infected with CVB1-conn5 (henceforth called CVB1), and Vero cells with wt CVB3, CVB4-E2 (henceforth called CVB4), and Mt10 as described previously [17,20]. In brief, Mt 10 vaccine virus was derived using a CVB3-infectious clone, pBRCVB3, developed previously in our laboratory [17]. The in vitro transcribed RNA from the infectious clone was used to propagate the virus in Vero cells in EMEM containing 10% fetal bovine serum (FBS). After ascertaining the cytopathic effects (CPEs), culture supernatants containing the virus were harvested, and viral stocks were stored in aliquots at −80 °C until further use. For viral titration, tissue culture infective dose 50 (TCID)_50_ values were determined using the Spearman–Karber method [21].

### 2.4. Challenge Studies

For vaccine studies, vaccine virus stock was diluted in 1x phosphate-buffered saline (PBS) to contain 0.5 × 10^6^ TCID_50_/200 µL, and the inocula were administered intraperitoneally (i.p.) into the mice (n = 18). Control (uninfected) mice (n = 15) received only 1x PBS. Groups of 2–3 mice were housed in filter-top cages assembled with closed air circulation. Cages containing the chow diet and waterers were changed every two days until the end of the experiment. Animals had access to food and water ad libitum and were inspected twice daily; body weights were recorded every two days. An alternative food and fluid source, trans gel diet (ClearH_2_O, Portland, ME, USA), was placed on the cage floor as needed. Sera were collected on days 0, 14, 21, and at termination on day 35. For challenge studies, mice were randomly assigned and administered with the vaccine virus (0.5 × 10^6^ TCID_50_/mouse diluted in 200 µL of 1x PBS, i.p., n = 26) or 1x PBS for control mice (200 µL, i.p., n = 10) on day 0. After 14 days, animals were injected with saline or CVB4 [2000 TCID_50_/mouse diluted in 200 µL of 1x PBS] i.p., and body weights were recorded every two days [17]. Hearts and pancreata were collected from animals that died naturally. At termination (21 days post-challenge), animals were euthanized, and serum, spleens, lymph nodes, hearts, and pancreata were collected for further analysis.

### 2.5. Histopathology

Hearts and pancreata were fixed by immersion in 10% phosphate-buffered formalin. Each tissue was sliced, and three representative 5 µm cross-sections were stained with hematoxylin and eosin (H and E). The sections were blinded to treatment and examined by a board-certified pathologist. Pathology scores were generated by enumerating inflammatory foci, necrosis, mineralization, and fibrosis. Individual scores were used to compare the qualitative nature of the lesions. Total scores represented the total foci of pathologic change across the three sections of the heart. Multiple changes present in a single focus were counted as 1 in the total count. The severity of pancreatic change was estimated as the percent of tissue section involvement from one random section of the pancreas. The nature of pancreatic lesions was noted as atrophy, inflammation, mineralization, necrosis, or a combination of these [17,22,23].

### 2.6. Virus Neutralization Assay

A virus neutralization test was performed using the sera obtained from different groups. LLC-MK2 (for CVB1) or Vero cells (for CVB3 and CVB4) were plated at 0.25 × 10^6^ cells/mL in 96-well plates to obtain 90–100% confluency. Serum samples were heat-inactivated at 56 °C for 30 min before testing, followed by two-fold serial dilutions (1:10 to 1:20,480). Equal volumes of CVB1, CVB3, or CVB4 suspension containing 100 TCID_50_/mL were incubated with serially diluted sera at 37 °C for one hour in a humidified chamber with 5% CO_2_. After incubation, 100 µL of the mixture of each dilution was added in duplicates to plates containing monolayers of cells and incubated at 37 °C. After four days, plates were observed for CPE, and the highest serum dilution that showed protection from CPE was determined to be the neutralization titer, and geometric mean titers (GMT) were calculated.

### 2.7. MHC-II Tetramer Staining

We have created major histocompatibility complex (MHC) class II/IA^k^ tetramers and dextramers to enumerate the frequencies of antigen-specific CD4 T cells by flow cytometry [17,24]. In this study, we used IA^k^ tetramers for VP1 721-740 and their corresponding controls (RNase 43-56) to detect virus-specific T cells. Briefly, single-cell suspensions of lymphocytes were obtained from various groups (saline, vaccine alone, CVB4 alone, vaccine/CVB4 challenge) 21 days post-challenge. Cells were stimulated with VP1 721-740 (20 µg/mL) for two days, and viable cells were maintained in a medium containing IL-2 [17,24]. During the 7–10 days post-stimulation, cells were stained with VP1 tetramers, and their controls as described above, followed by anti-CD4 (GK1.5, BioLegend, San Diego, CA, USA) and 7-aminoactinomycin D (7-AAD, Invitrogen, Carlsbad, CA, USA) [25,26,27]. After cells were acquired by flow cytometry (FACSCalibur, BD Biosciences, CA, USA), percentages of tetramer-positive cells were determined in the live (7-AAD^¯^) CD4^+^ subset using FlowJo software [v 7.6.5] (Tree Star, Ashland, OR, USA) [24,28].

### 2.8. Determination of CVB-Reactive Antibodies

Serum samples from various groups (saline, vaccine alone, CVB4 alone, vaccine/CVB4 challenge) were collected on days 0, 14, and 35 and analyzed for total Ig, IgG1, IgG2a, IgG2b, IgG3, IgA, IgM, and IgE as described previously [17]. In brief, 96-well polystyrene microtiter plates were coated with or without CVB3 VP1 or an irrelevant control (KLH; eBioscience, San Diego, CA, USA) (5 μg/mL) in 1x coating buffer and incubated at 4 °C overnight. Plates were washed with 1x PBS/0.05% Tween-20 and blocked with 1x PBS/2% BSA/5% normal goat serum for 1.5 h at RT (1:150); serum samples were then added in duplicates. Plates were incubated at 37 °C for 1 h and washed. Horseradish peroxide (HRP)-labeled goat anti-mouse IgA, IgE, IgM, IgG1, IgG2a, IgG2b, IgG3, and total Ig (Southern Biotech, Birmingham, AL, USA) were added as secondary antibodies. After the plates were incubated at RT for 2 h, 1x tetramethylbenzidine solution (eBioscience) was added as a substrate, and reactions were stopped using 1M phosphoric acid. Plates were read at 450 nm using an automated ELISA reader (BioTek Instruments, Winooski, VT, USA), and optical density (OD) values were measured. Additionally, where indicated, measurement of total immunoglobulins (Igs) and their isotypes (IgG1, IgG2a, IgG2b, IgG3, IgA, and IgM) was performed on serum samples using the LEGENDplex Murine Ig isotyping panel (6-plex; BioLegend).

### 2.9. Cytokine Analysis

Cytokines were measured using either sera obtained from saline and vaccine recipients, with or without CVB4 challenge or day 3 culture supernatants of lymphocyte cultures prepared from the above groups stimulated with or without VP1 681-700 and VP1 721-740 (20 μg/mL). Cytokine analysis was performed using the LEGENDplex Murine Th cytokine Panel (12-plex; BioLegend). The panel included IL-2, IFN-γ, IL-4, IL-5, IL-6, IL-9, IL-10, IL-13, IL-17A, IL-17F, IL-22, and tumor necrosis factor (TNF)-α. Standard curves were obtained by serially diluting the lyophilized mouse cytokine standard mix provided in the kit. Briefly, capture bead/cytokine antibody conjugates were first prepared, and the mixtures were added to a tube containing diluted standards or test samples, followed by the addition of streptavidin-phycoerythrin detection antibodies. After acquisition by flow cytometry, cytokine concentrations were determined using the LEGENDplex data analysis software suite (BioLegend) [17,29].

### 2.10. RNA Isolation and Real-Time Quantitative PCR

Hearts and pancreata stored at −80°C were used for RNA isolation. Approximately 20–30 mg of tissue was transferred to the RLT buffer and homogenized with a FastPrep96 system as recommended (Lysing Matrix D 1.4-mm ceramic beads; MP Biomedicals, Irvine, CA, USA). RNA was isolated using the RNeasy kit (Qiagen, Hilden, Germany); samples were treated with deoxyribonuclease (DNase) I and quantified using the NanoDrop ND-1000 spectrophotometer (Thermo Fisher Scientific, Waltham, MA, USA). In a single-step reaction, RNA was reverse-transcribed, and PCR was performed using the iTaq Universal one-step RT-qPCR kit (BioRad, Hercules, CA, USA). The real-time quantitative PCR analysis included amplifications for CVB3 VP1 (target gene) and glyceraldehyde-3-phosphate dehydrogenase (GAPDH, housekeeping gene) using TaqMan Gene Expression Assays (Applied Biosystems) and the CFX96 Touch Real-time PCR detection system (BioRad). Expression of CVB3 VP1 was normalized to GAPDH using the 2^−(ΔΔ*C*t)^ method as reported previously [17].

### 2.11. Statistical Analysis

Statistical analyses were performed using GraphPad Prism software v8.0 (GraphPad Software, Inc., La Jolla, CA, USA). Data sets on tetramer^+^ cells, antibodies, immunoglobulins, cytokines, body weights, and qPCR were analyzed by unpaired Student’s *t*-test, Mann–Whitney test, two-way ANOVA with Sidak’s post-test, or Kruskal–Wallis test for pairwise comparisons between the groups. Log-rank test with Bonferroni correction was used to analyze the statistical significance of the survival curves. Barnard’s exact test was used to analyze the histological parameters. Graphs were prepared by GraphPad Prism software v8.0.

## 3. Results and Discussion

We recently reported that a novel live attenuated CVB3 vaccine virus, Mt10, produces high nAB titers and provides complete protection against CVB3-induced myocarditis and pancreatitis [17]. Using this vaccine virus, we sought to determine its ability to generate cross-protection against other serotypes of CVB. Essentially, the vaccine virus was created by introducing an alanine mutation for histidine at amino acid position 790 in the VP1 region of the CVB3 viral canyon. In our study of the structural characteristics of the virus-CAR-interacting region, we noted histidine to be a buried residue, the mutation of which did not alter the ability of the vaccine virus to infect cells [17]. Thus, the use of the Mt10 virus as a vaccine candidate offered the advantage of infecting cells and generating protective immune responses without inducing the disease. We expanded this observation to test the hypothesis that the vaccine virus could generate effective immune responses for closely related CVB serotypes by cross-reactivity.

### 3.1. Vaccine Virus Induces nABs against Both CVB1 and CVB4

**Figure 1:**
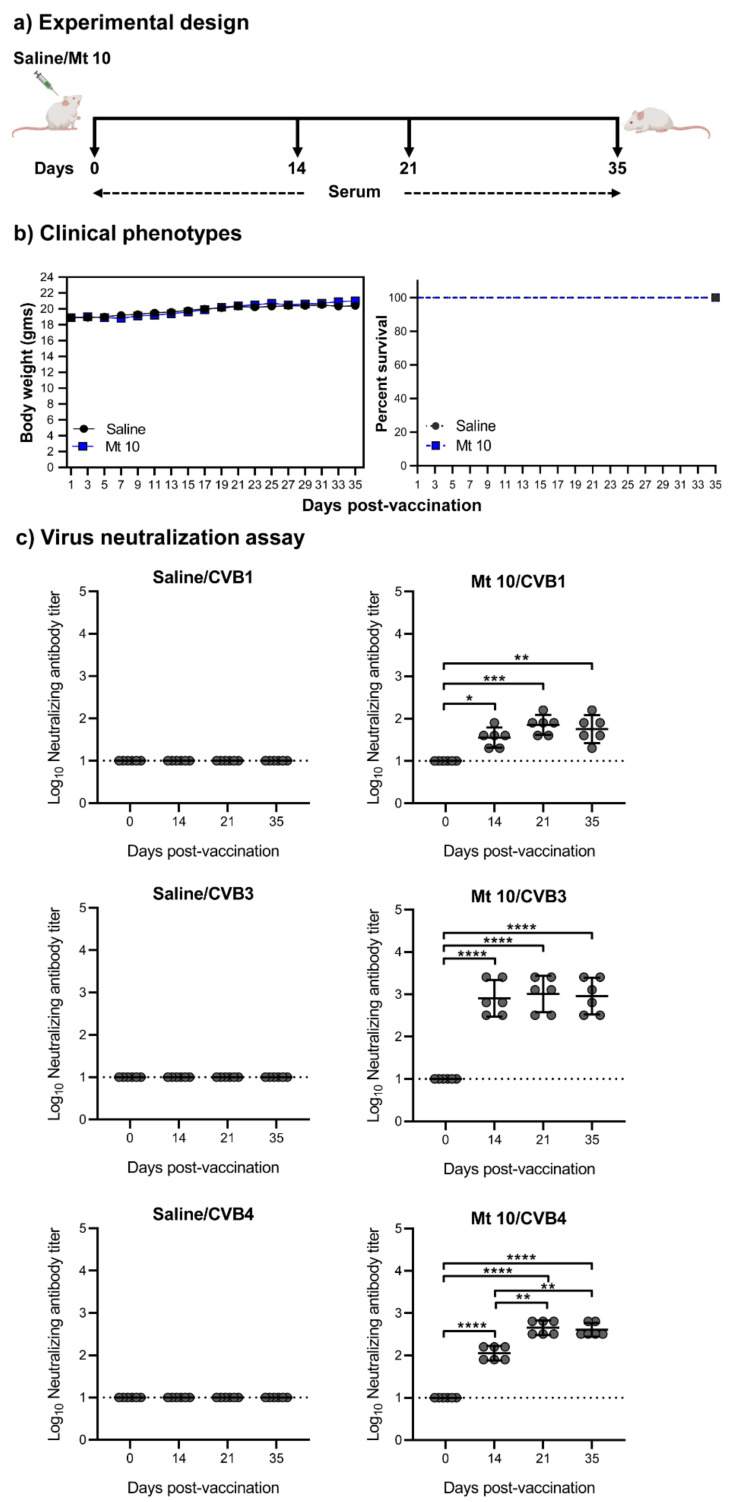
Vaccine virus produces nABs against multiple CVB serotypes. (**a**) Experimental design. A/J mice were vaccinated with Mt10 virus (n = 18); saline recipients served as controls (n = 15). Sera were collected on days 0, 14, 21, and 35 post-vaccination and terminated on day 35. (**b**) Clinical phenotypes. Body weights were measured once every two days until termination and compared between groups (left panel), and survival rates were tabulated (right panel). (**c**) Virus neutralization assay. Sera were obtained from saline and vaccine recipients on days 0, 14, 21, and 35, serial dilutions up to 1:20,480 were made, and samples were incubated with CVB1 (top panel), CVB3 (middle panel), or CVB4 (bottom panel). The mixtures were later transferred to plates containing monolayers of LLC-MK2 cells for CVB1 or Vero cells for CVB3 and CVB4 and incubated for four days to calculate the percentage neutralization based on CPE caused by the viruses. Data from n = 6 samples, each representing a pool of sera from 3 to 5 mice, are shown. Two-way ANOVA with a Sidak’s post-test was used to compare nABs and body weight changes in the saline group relative to the vaccine-recipient group; log-rank test with Bonferroni correction was used to compare survival curves. * *p* ≤ 0.05, ** *p* ≤ 0.01, *** *p* ≤ 0.001, and **** *p* ≤ 0.0001.

To evaluate cross-protective immune responses generated by the vaccine virus, we considered two serotypes, CVB1 and CVB4. While CVB3 is generally implicated in the causation of myocarditis [4,30], CVB4 and CVB1 infections were shown to be associated with T1D [13,31,32,33], and all three serotypes can induce pancreatitis [5,24,34]. We immunized groups of mice with or without vaccine virus and collected sera on days 0, 14, 21, and 35 post-vaccination (Figure 1a). To determine the clinical phenotypes and the effect of the vaccine virus on mice, body weights were measured every two days, and mortalities, if any, were noted. Figure 1b, left panel indicates that the vaccine recipients did not lose body weight throughout the study. Expectedly, animals in both the vaccine and saline groups were clinically healthy, and no mortalities were noted (Figure 1b, right panel). We next determined the protective effect of the vaccine virus against CVB1, CVB3, and CVB4 by analyzing the nAB titers in the sera obtained from vaccinated animals. As indicated in Figure 1c, left panels, sera from saline recipients did not contain nABs against CVB1, CVB3, or CVB4 at all time points of the study, leading us to set the 1:10 serum dilution as the baseline. Contrastingly, the vaccine sera revealed nABs with GMT of 36 for CVB1 on day 14, with an upward trend seen from day 14 to day 35 with a GMT up to 72 (Figure 1c, top right panel). We also analyzed the nAB titers against CVB3 (Figure 1c, middle right panel). Expectedly, we noted significantly higher GMTs of 806, 1016, and 905 on days 14, 21, and 35, respectively, when compared to the saline recipients [17] (Figure 1c, middle panel). Similarly, the nAB titers for CVB4 obtained from day 14 vaccine sera had a GMT of 113, and the antibody titers were further enhanced on day 21 (GMT 453) and remained elevated up to day 35 with GMT of 403 (Figure 1c, bottom right panel). Furthermore, a four-fold increase in nAB titers was noted for CVB4 on days 21 and 35 when compared to day 14 (*p* ≤ 0.01) (Figure 1c, bottom right panel), indicating a gradual increment in nABs against CVB4.

Of note, comparisons of viral proteome sequences revealed 91% similarity between vaccine virus and CVB1 and 88% similarity between the vaccine virus and CVB4. The finding that the nAB titers were greater for CVB4 than for CVB1 suggests that the high degree of similarity between serotypes does not necessarily mean that the induction of cross-reactive immune responses may ensue. Additionally, nAB titers in the lower range, as is in our case for CVB1 (GMT from 50 to 70), does not necessarily equate to the absence of protective effects, as infections with lower nAB titers (1:10 for SARS-CoV-2; 1:40 for Influenza) have also been shown to be protective [35,36].

### 3.2. The Vaccine Virus Protects against CVB4 in Challenge Studies

**Figure 2:**
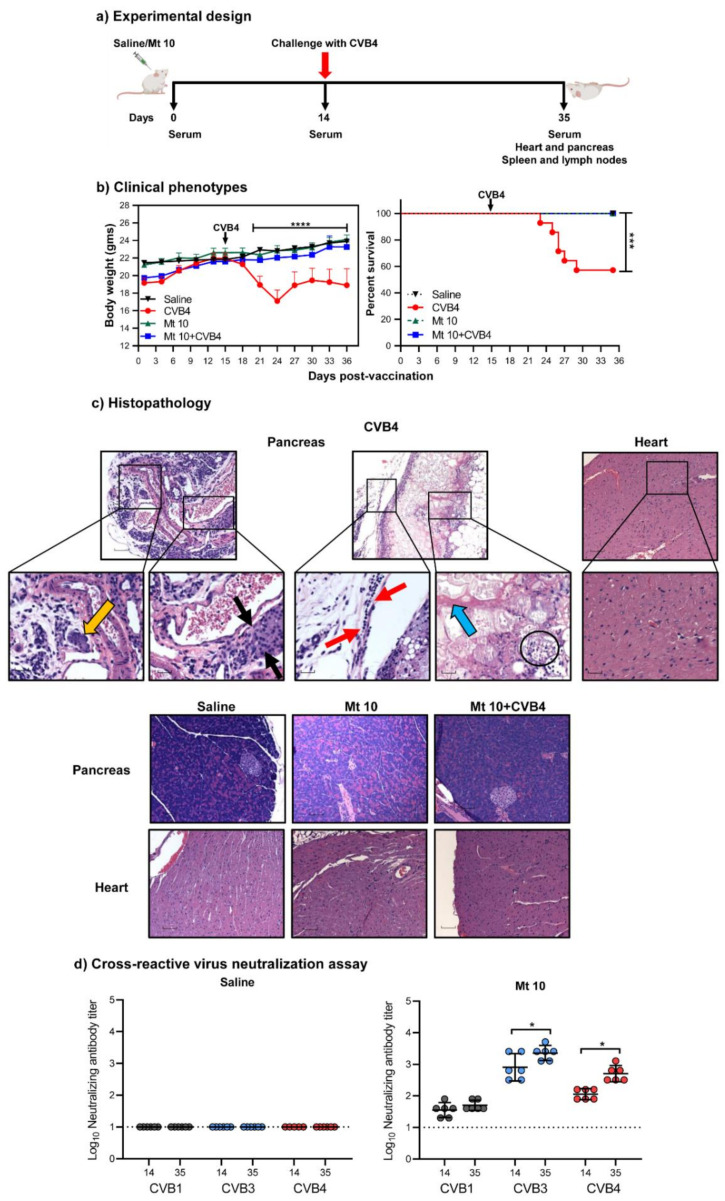
Vaccine virus offers cross-protection against CVB4 infection in challenge studies. (**a**) Experimental design. Two groups of mice each were given saline or vaccinated with the vaccine virus on day 0 after the serum was collected. After 14 days, serum was collected again, and one group from each treatment was challenged with CVB4. Experiments were terminated 21 days post-challenge, and serum and tissues were collected for in vitro experimentation. (**b**) Clinical phenotypes. Body weights (left panel) and survival rates (right panel) of different groups are shown. (**c**) Histopathology. Hearts and pancreata were collected from the indicated groups and processed by H and E staining to evaluate for any inflammatory changes. Representative pancreatic and heart sections are shown. Pancreatic sections and their insets from the CVB4 group had a cluster of acinar cells (orange arrow) and showed atrophy (black arrows), lymphocytic infiltrates (red arrow), necrosis (blue arrow), and karyorrhexis debris from necrotic adipocytes (circle), as opposed to normal sections in the saline, vaccine, and vaccine/CVB4-challenged groups. Magnification, 20×; scale bars, 20 µm. Data sets obtained from three individual experiments, each involving n = 3–8 mice, are shown. (**d**) Cross-reactive virus neutralization assay. Sera were collected from saline and vaccine/CVB4-challenged groups on days 14 and 35, and virus neutralization assays were performed on LLC-MK2 (for CVB1) or Vero cell (for CVB3 and CVB4) monolayers. Incubation was continued for four days to calculate the nAB titers based on CPE. Data from n = 6 samples, each representing a pool of sera from 3 to 5 mice, are shown. Two-way ANOVA with a Sidak’s post-test was used to compare body weight changes in the saline, vaccine, and vaccine/challenged groups relative to the CVB4 group; log-rank test with Bonferroni correction was used to compare survival curves. * *p* ≤ 0.05, *** *p* ≤ 0.001, and **** *p* ≤ 0.0001.

We recently established the CVB4 infection model in A/J mice and noted that CVB4 could induce severe pancreatitis similar to the severity of disease induced by CVB3, but the incidence of myocarditis was absent or infrequent [24]. We had also identified T cell epitopes common to both CVB3 and CVB4 [24]. Since the CVB4 mouse model was available and the vaccine virus also induced robust cross-reactive nAB responses as compared to CVB1, we were prompted to evaluate the protective effects of our vaccine virus. We vaccinated groups of mice with a single dose of vaccine virus or saline on day 0 and challenged them 14 days later with CVB4 (Figure 2a). After 21 days, animals were euthanized, and tissues and sera were collected for histology and serological assays, respectively. Clinically, recipients of CVB4 alone started to lose bodyweight ~4 days post-infection and did not regain the weight thereafter (*p* ≤ 0.0001) (Figure 2b, left panel). Conversely, vaccine virus recipients that were challenged with CVB4 showed no reduction in body weight as compared to the saline controls. Likewise, a 43% mortality rate (6/14) was noted in the CVB4-alone group (*p* ≤ 0.001) (Table 1 and Figure 2b, right panel), but not in other groups, suggesting that the vaccine virus prevented the infection. To further support this observation, we evaluated hearts and pancreata for inflammatory changes, leading us to note pancreatitis in 86% of animals in the CVB4-alone group, with atrophy (86%) and inflammation (71%) as the dominant features, but necrosis was less frequently noted (14%) (Table 1 and Figure 2c). One animal (1/12, 8%) in each of the vaccine-alone and CVB4-challenged groups had mild atrophy and inflammation in the pancreas. Nonetheless, myocarditis was not found in any of the above groups, and, expectedly, neither myocarditis nor pancreatitis was noted in the saline group. Furthermore, VNT from sera collected on day 14 before challenge with CVB4 and day 35 from vaccinated mice challenged with CVB4 revealed nABs to be present for all three serotypes in the order of CVB3 > CVB4 > CVB1 when compared to saline recipients (Figure 2d). We noted the GMTs for CVB3 on day 35 to be ~three-fold (GMT of 2281) more than day 14 before challenge with CVB4 (GMT of 806) (*p* ≤ 0.05), and that of CVB4 on day 35 to be ~five-fold (GMT of 508) more than day 14 (GMT of 113) (*p* ≤ 0.05). These data suggest that exposure to CVB4 in vaccinated mice can augment the nAB titers. Taken together, vaccination with the Mt10 virus followed by challenge with another CVB serotype could induce cross-protective nABs against multiple CVB serotypes, pointing to the possibility that the protection mediated by the vaccine virus may involve the generation of antigen-specific immune responses in vivo. 

### 3.3. Virus-Reactive Antibody Responses Revealed the Generation of Mainly IgG Isotypes

To evaluate antibody responses, we initially measured total concentrations of various antibody isotypes independent of antigen specificity. In this setting, serum was collected from the four groups on the days indicated in parentheses: saline (days 0, 14, and 35); vaccine-alone (day 14 and 35); and vaccine/CVB4 challenge and CVB4-alone, the two groups in which CVB4 infection was induced on day 14 post-vaccination (day 21 post-infection, indicated as day 35) (Figure S1). The analysis revealed no antibodies in the saline group, whereas the vaccine group, in comparison with saline recipients, had increased concentrations of IgG1 (*p* ≤ 0.01) and IgG2b (*p* ≤ 0.05). In contrast, the CVB4-alone group showed significant elevations of IgG2a (*p* ≤ 0.05), IgG2b (*p* ≤ 0.05), IgG3 (*p* ≤ 0.0001), and IgA (*p* ≤ 0.0001) (Figure S1). These observations suggest that CVB4 infection might have boosted the vaccine-induced IgG2a response. Conversely, elevated IgG3 and IgA levels might have occurred specifically in response to CVB4 infection. However, to understand the significance of these antibodies, additional studies were needed to evaluate their antigen specificity.

**Figure 3:**
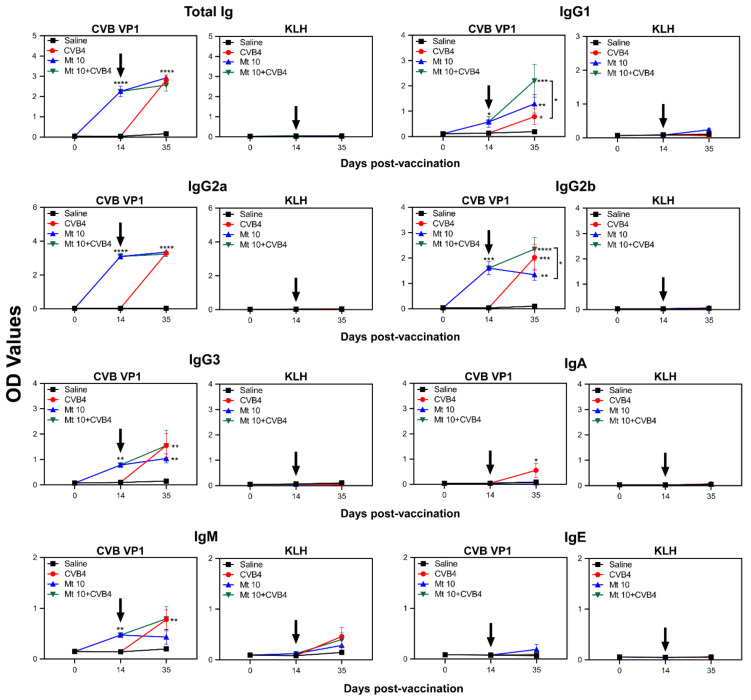
Vaccinated mice induce VP1-specific nABs. Serum samples collected from the indicated groups at various time points were diluted (1:150) and added in duplicates to high-binding plates previously coated with CVB3-VP1 or KLH (control). After adding HRP-conjugated goat anti-mouse total Ig, IgG1, IgG2a, IgG2b, IgG3, IgA, IgM, and IgE as detection antibodies, reactions were stopped. Plates were read at 450 nm to obtain the OD values. Mean ± SEM values obtained from n = 6 samples, each representing 3 to 5 mice, are shown. Two-way ANOVA and Mann–Whitney test were used to determine significance between groups. * *p* ≤ 0.05, ** *p* ≤ 0.01, *** *p* ≤ 0.001, and **** *p* ≤ 0.0001.

To that end, we analyzed virus-reactive antibodies using VP1 as a specific antigen and KLH as an irrelevant control using the groups described above (Figure 3). As expected, antibody reactivity was lacking in control groups (saline and KLH). The vaccine-alone group revealed VP1-reactivity for mainly total IgG, IgG1, IgG2a, IgG2b, and IgG3, but reactivity for IgM was low, as we have previously demonstrated [17]. By analyzing antibodies on day 21 post-challenge in the CVB4-alone and vaccine/CVB4 challenge groups, we made two major observations: (i) IgG2b was significantly elevated in the vaccine/challenge group compared to the vaccine alone group (*p* ≤ 0.05) and to a lesser extent, IgG1, IgG3, and IgM, suggesting that CVB4 infection might have boosted the vaccine-induced IgG2b response. These observations may suggest the possible existence of epitopes common to both CVB3 and CVB4, and their identification may create opportunities to develop subunit vaccines as demonstrated for CVB3, Enterovirus 71, and SARS-CoV-2 [37,38]. However, such an effect was not observed for IgG2a. (ii) Determination of antibody-reactivity indices between various isotypes on day 21 post-challenge (Figure 3) for IgG1/IgG2a (0.62 for vaccine/CVB4 challenge; 0.38 for vaccine group; 0.25 for CVB4 alone) and IgG2b/IgG2a (0.72 for vaccine/CVB4 challenge; 0.44 for vaccine group; 0.66 for CVB4-alone) indicate that IgG2a and IgG2b generated may be critical for disease protection. Overall, relating the patterns of antibody isotypes based on the measurement of total amounts independent of antigen specificity (Figure S1) with those of VP1-reactivity (Figure 3) suggest that both profiles complement each other. Thus, measurement of antibody isotypes in the absence of availability of specific recombinant proteins may still be informative to predict the nature of antigen-specific responses. Further, since IgG isotypes formed a major component of antibody response, our data suggest the possibility that antigen-specific T cells might have been involved in antibody production.

### 3.4. VP1-Specific T-Cell Responses Generated in Vaccinated and Challenged Animals Predominantly Induce Th1 Cytokines

**Figure 4:**
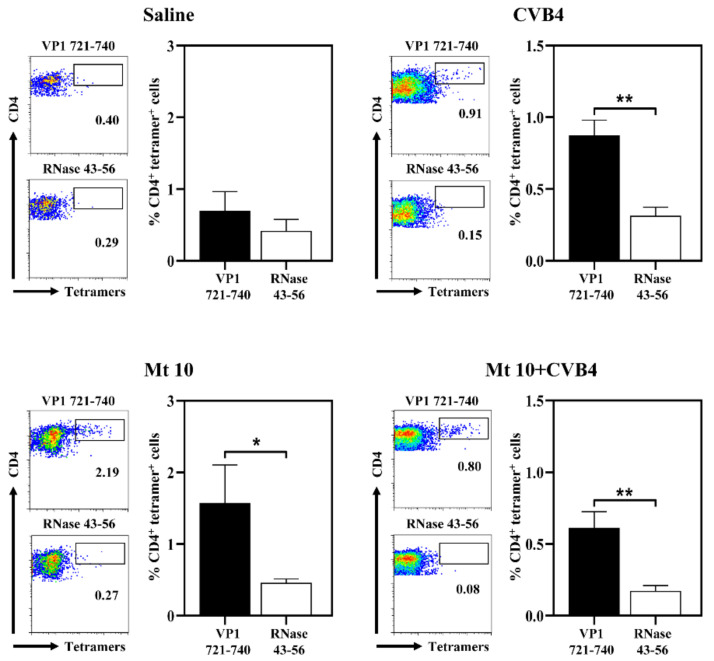
Vaccinated mice challenged with CVB4 induce the generation of VP1-specific CD4 T cells. Lymphocytes were prepared from the indicated groups as described in the methods. Cells were stimulated with VP1 721–740 and maintained in IL-2 medium supplemented with antibiotics. Viable cells were harvested between 7 and 11 days post-stimulation and were stained with the indicated IA^k^ tetramers, anti-CD4, and 7-AAD. After cells were acquired by flow cytometry, tetramer^+^ cells were analyzed in the live (7-AAD¯) CD4^+^ subset using FlowJo software. RNase 43–56, control. Representative flow cytometric dot-plots and the mean ± SEM values from three individual experiments, each involving n = 3–6 mice, are indicated. Unpaired Student’s *t*-test (two-tailed) was used to determine significance between treatment groups. * *p* ≤ 0.05 and ** *p* ≤ 0.01.

First, we sought to determine antigen-specific T-cell responses using MHC class II/IA^k^ tetramers that we recently generated for VP1 721–740 [17,24]. Using these reagents, we analyzed the frequencies of virus-specific CD4 T cells by flow cytometry, with RNase 43-56 serving as control tetramers (Figure 4).

Lymphocytes were prepared from saline and vaccine groups on day 35 and from CVB4 and vaccine/CVB4-challenged groups on day 21 post-infection. Cells were stimulated with VP1 721–740, and after resting cells in IL-2 medium, tetramer staining was performed [17,24]. Expectedly, lymphocytes generated from the saline group did not reveal any significant binding to VP1 721–740 tetramers (Figure 4). Conversely, cells generated from other groups (vaccine-alone: 1.6 ± 0.5; *p* ≤ 0.05; CVB4-alone: 0.8 ± 0.2; *p* ≤ 0.01; and vaccine/CVB4-challenged: 0.6 ± 0.1; *p* ≤ 0.01) revealed significant staining to VP1 721–740 tetramers as compared to control tetramers (Figure 4). Of note, sequence comparison of VP1 721–740 between CVB3 and CVB4 revealed a similarity of 90% (CVB3: PDKVDSYVWQTSTNPSVFWT and CVB4: PTKVDDYVWQTSTNPSVFWT; similar residues are underlined) [24]. The finding that CVB4-sensitized lymphocytes were stained with the VP1 721–740 tetramers suggests that the minor variations at the N-terminal end did not preclude tetramers from binding the antigen-specific T cells. However, we had expected tetramer staining to be higher in the vaccine/challenged group than the vaccine group, but this was not the case. This variation may be due to differences in the sampling days as described above. Nonetheless, it is possible that enhanced T-cell responses still might have been evident in the vaccine/challenged group for epitopes other than VP1 721–740 and those representing other structural and non-structural VPs, which we have not investigated in our studies. Therefore, the use of additional readouts such as cytokines might be helpful in analyzing antigen-specific T-cell responses.

**Figure 5:**
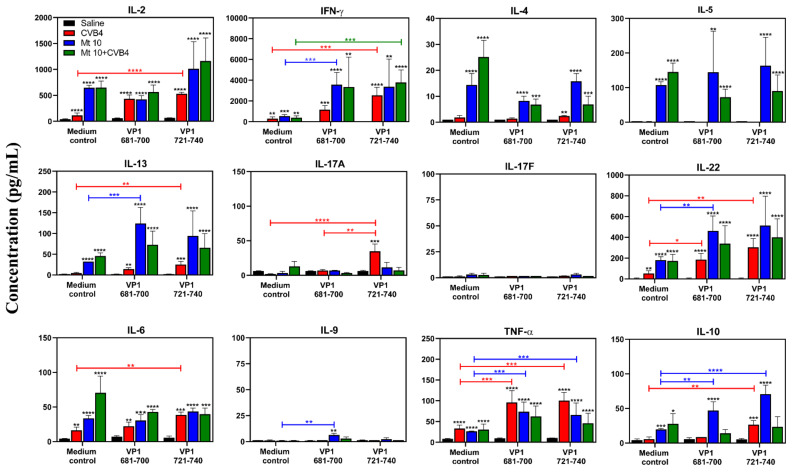
T-cell responses induced in mice vaccinated and infected with CVB4 were skewed towards mainly the Th1 phenotype. Supernatants collected on day 3 post-stimulation with or without peptides (VP1 681–700, VP1 721–740) from the indicated groups were analyzed for cytokines using LEGENDplex cytokine bead array as described in the methods section. Mean ± SEM values obtained from three individual experiments, each involving n = 3–6 mice, are indicated. Unpaired Student’s *t*-test (two-tailed) or two-way ANOVA was used to determine significance between various treatment groups. * *p* ≤ 0.05, ** *p* ≤ 0.01, *** *p* ≤ 0.001, and **** *p* ≤ 0.0001. Black asterisk indicates *p* values from the comparison of treatment groups with the saline group.

To analyze cytokines, we adopted the LEGENDplex bead array analysis, permitting us to evaluate multiple cytokines under uniform conditions in lymphocyte culture supernatants and serum involving the four groups (saline, vaccine, CVB4, and vaccine/CVB4-challenged). To obtain culture supernatants, lymphocytes generated from these groups were stimulated with or without VP1 681-700 or VP1 721-740. On day 3 post-stimulation, supernatants were harvested for Th1 (IL-2, IFN-γ), Th2 (IL-4, IL-5, IL-13), Th17 (IL-17A, IL-17F, IL-22), and other inflammatory (IL-6, IL-9, TNF-α) or anti-inflammatory cytokines (IL-10). The major findings were: (i) Supernatants from the saline group cultured in the absence of viral peptides did not reveal any of the cytokines tested (Figure 5).

However, supernatants obtained from other groups (vaccine, CVB4, and vaccine/CVB4) in the absence of peptide stimulations (medium control) showed elevated amounts of IL-2, IFN-γ, IL-4, IL-5, IL-13, IL-22, IL-6, TNF-α, and IL-10, but not IL-17A, IL-17F, and IL-9. Such a pattern occurred consistently in vaccine and vaccine/CVB4-challenged groups compared to the CVB4 group suggesting that the vaccine-induced T-cell responses may still be in the effector state in vivo, leading to spontaneous secretion of cytokines in cell cultures. (ii) Comparison of cytokines between VP1 681–700 treatment and its medium control for the vaccine group revealed elevated levels of IFN-γ (*p* ≤ 0.001), IL-13 (*p* ≤ 0.001), IL-22 (*p* ≤ 0.01), TNF-α (*p* ≤ 0.001), and IL-10 (*p* ≤ 0.01). Similar elevations also were noted with VP1 721–740 for TNF-α (*p* ≤ 0.001) and IL-10 (p ≤ 0.01). (iii) Cytokine analysis in the CVB4 group revealed profiles similar to those in the vaccine group, and increased amounts were noted for IL-2 (*p* ≤ 0.0001), IFN-γ (*p* ≤ 0.001), IL-13 (*p* ≤ 0.01), IL-17A (*p* ≤ 0.0001), IL-22 (*p* ≤ 0.01), IL-6 (*p* ≤ 0.01), TNF-α (*p* ≤ 0.001), and IL-10 (*p* ≤ 0.01) in culture supernatants obtained from VP1 721–740. Similar elevations were noted for IL-22 (*p* ≤ 0.05) and TNF-α (*p* ≤ 0.001) in culture supernatants obtained from VP1 681–700. (iv) When the amounts of cytokines produced in vaccine and vaccine/challenged groups were compared, we noted that none of the cytokines were elevated. It may be that exposure to CVB4 in vaccine recipients might not have modulated the vaccine-induced T-cell responses, raising a question of whether the protection induced by the vaccine virus may be primarily due to virus-reactive antibodies. Alternatively, antibodies produced in response to the vaccine virus before challenge might have rapidly cleared the virus to be able to further trigger T-cell responses.

We then addressed whether the cytokines detected in lymphocyte cultures can be correlated with the serum cytokines by analyzing the cytokines in serum samples harvested on day 14 post-vaccination or day 21 post-challenge in different groups and compared them with the saline group (Figure S2). The analysis indicated significant elevations in IL-22 (*p* ≤ 0.001), IL-6 (*p* ≤ 0.0001), and TNF-α (*p* ≤ 0.05) in vaccine/challenged animals as compared to other groups (Figure S2). However, a relative increase in IFN-γ (*p* ≤ 0.05) production was seen in the CVB4 group as compared to other treatments. The finding that IL-22 was secreted in higher concentrations in vaccinated/vaccine-challenged groups (Figure 5) may indicate a beneficial role for IL-22 in protection against CVB4-infection, as demonstrated in the pancreatitis model in rats [39]. Furthermore, in culture supernatants (Figure 5), but not in serum (Figure S2), IL-10 was found to be increased in the vaccine group, and to a lesser extent in the CVB4 group that may have anti-viral effects as demonstrated in CVB3 and other infections [40,41,42]. Although similarities exist between the cytokine profiles of lymphocyte culture supernatants and serum, the former reflects antigen-specific responses that could be reliably used as indicators of T-cell responses. Overall, by comparing the quantity of cytokines produced in various treatment groups, we noted that IFN-γ was found to be the dominant cytokine produced in large amounts as seen in CVB4-infected patients [43], which also correlated with the increased secretion of IgG2a (Figure 3), implying that IFN-γ might be critical for vaccine-induced protection.

### 3.5. Vaccinated Mice Are Cleared of Viral RNA Post-Challenge with CVB4

**Figure 6:**
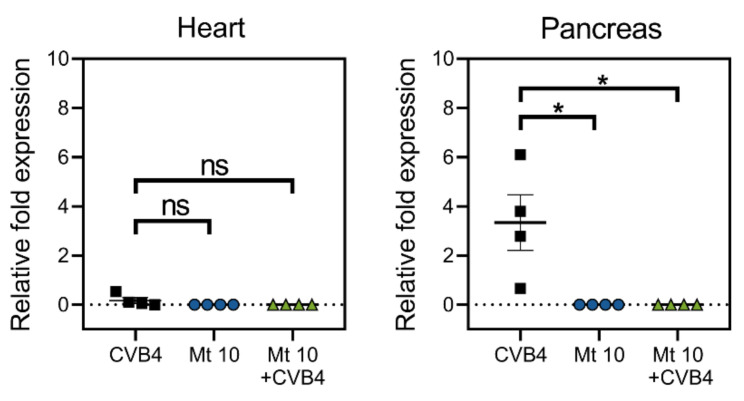
Vaccinated mice challenged with CVB4 did not reveal detection of viral nucleic acids. Groups of mice were given saline or were vaccinated with Mt10 virus, and 14 days later, challenged with or without CVB4. Hearts and pancreata were collected three weeks post-challenge, total RNA was extracted, and viral RNA was estimated by qPCR targeting VP1 sequences. After normalizing the expression levels of viral RNA relative to GAPDH, 2^−(ΔΔCt)^ values were calculated. Mean ± SEM values representing four samples per group, each containing n = 3–5 mice, are shown. Mann–Whitney test was used to determine significance between groups. * *p* ≤ 0.05; ns—not significant.

To evaluate the efficacy of the vaccine virus for its ability to prevent infection, we sought to evaluate if vaccinated mice challenged with CVB4 had any residual virus post-challenge. We examined hearts and pancreata from saline, vaccine, CVB4, and vaccine/CVB4-challenged groups to detect viral nucleic acid (Figure 6). Since the VP1 of CVB3 and CVB4 had high sequence similarities (81%), we used the CVB3 VP1 to quantify the viral RNA present in the hearts and pancreas by qRT-PCR. As shown in Figure 6, no viral RNA was detected in the hearts of any of the treatment groups that included the saline group. However, as expected, the pancreas from CVB4-infected mice had viral nucleic acid present (*p* ≤ 0.05), whereas animals from the vaccine group or those challenged with CVB4 had no viral RNA. Thus, our data suggest that the vaccine virus-induced cross-protection against CVB4 infection might have involved a combination of virus-reactive antibodies and T-cell responses.

In summary, we have demonstrated that a single dose of vaccine virus generated on the CVB3 backbone induces cross-reactive nABs against related serotypes, namely CVB1 and CVB4, but the depth was striking against CVB4. In vivo challenge studies with CVB4 indicated that the cross-protection was found to be 92% efficacious with no residual virus detected in the vaccine recipients. Furthermore, the antibodies generated after CVB4 challenge retained neutralizing ability against CVB1, CVB3, and CVB4. Mechanistically, the production of VP1-specific antibodies, mainly IgG2a and IgG2b, and CD4 T cells producing IFN-γ, and IL-22 might have played critical roles in the vaccine-induced protection from CVB4 infection. Although various approaches (live, inactivated, virus-like particles) have been used to develop vaccine candidates for CVB3 [44,45,46,47] and CVB1 [48,49], limited data are available regarding their utility against cross-protection for CVB4. Additionally, studies that aimed to show cross-protection did not indicate the production of antigen-specific antibody and cytokine responses [50]. A recent report of a hexavalent-vaccine cocktail containing the formalin-inactivated viruses of all six serotypes was effective in inducing protective responses to CVB4, but it required a three-dose prime-boost strategy [51]. Our approach is unique in that the attenuated vaccine virus was derived based on its ability to retain the infectivity and immunogenicity without causing the disease, which offers an advantage that the vaccine virus can induce both humoral and cell-mediated responses. To study these parameters, we have developed novel tools, namely, MHC class II tetramers, that permitted us to capture virus-specific, CD4 T-cell responses, in addition to measuring the VP1-specific Ab responses. We envision a possibility that the Mt10 vaccine virus can potentially induce cross-protection to all CVB serotypes since the mutation introduced in the CAR-interacting region of the viral canyon is preserved in all of them. Thus, our work may pave the way for developing monovalent vaccines to prevent CVB infections caused by multiple serotypes.

## Figures and Tables

**Table 1:** Histological evaluation of hearts and pancreata in saline, CVB4, Mt 10-vaccinated, and Mt 10-vaccinated and challenged mice.

Parameters	Saline	CVB4	Mt 10	Mt 10+CVB4
**Heart**				
**Incidence**	0/10 (0.0)	0/14 (0.0)	0/12 (0.0)	0/12 (0.0)
**Mortality**	0/10 (0.0)	6/14 (42.8)	0/12 (0.0)	0/12 (0.0)
**Myocardial lesions**	0.0 (0.0)	0.0 (0.0)	0.0 (0.0)	0.0 (0.0)
**Pancreas**				
**Incidence**	0 (0.0)	12/14 (85.7)	1/12 (8.3)	1/12 (8.3)
**Atrophy**	0 (0.0) ^a^	12/14 (85.7)	1/12 (8.3) ^a^	1/12 (8.3) ^a^
**Inflammation**	0 (0.0) ^a^	10/14 (71.4)	0/12 (0.0) ^a^	1/12 (8.3) ^a^
**Necrosis**	0 (0.0)	2/14 (14.2)	0/12 (0.0)	0/12 (0.0)
**Mineralization**	0 (0.0)	0/14 (0.0)	0/12 (0.0)	0/12 (0.0)

() indicates percentages. ^a^ denotes significant differences in comparison with the CVB4 group (*p* < 0.0001).

## Data Availability

Not applicable.

## Supplementary Materials

The following are available online at https://www.mdpi.com/article/10.3390/microorganisms9112323/s1, Figure S1. Detection of serum Ig isotypes. Sera collected on various days from indicated groups were analyzed to quantify Ig isotypes using LEGENDplex bead array as described in the methods section. Mean ± SEM values obtained from three individual experiments, each involving n = 3–6 mice, are indicated. Black downward arrow indicates time point of CVB4 challenge. Two-way ANOVA and Mann–Whitney test were used to determine significance between groups. * *p* ≤ 0.05, ** *p* ≤ 0.01, *** *p* ≤ 0.001, and **** *p* ≤ 0.0001. Figure S2. Serum cytokine analysis of vaccinated mice. Sera collected on days 0, 14, and 35 post-vaccination from the indicated groups were analyzed for cytokines using LEGENDpleax cytokine bead array as described in the methods section. Mean ± SEM values obtained from three individual experiments, each involving n = 3–6 mice, are indicated. Black downward arrow indicates time point of CVB4 challenge. Two-way ANOVA was used to determine significance between various treatment groups. * *p* ≤ 0.05, ** *p* ≤ 0.01, *** *p* ≤ 0.001, and **** *p* ≤ 0.0001.
