# Supplementary material for: Mt10-CVB3 Vaccine Virus Protects against CVB4 Infection by Inducing Cross-Reactive, Antigen-Specific Immune Responses
Source: Microorganisms. 2021 Nov 10;9(11):2323. doi: 10.3390/microorganisms9112323 (PMC8622534; doi:10.3390/microorganisms9112323)
Supplement: Supplementary file 1 [file microorganisms-09-02323-s001.zip › microorganisms-1433348-supplementary.pdf]

microorganisms

MDPI

Supplementary Material

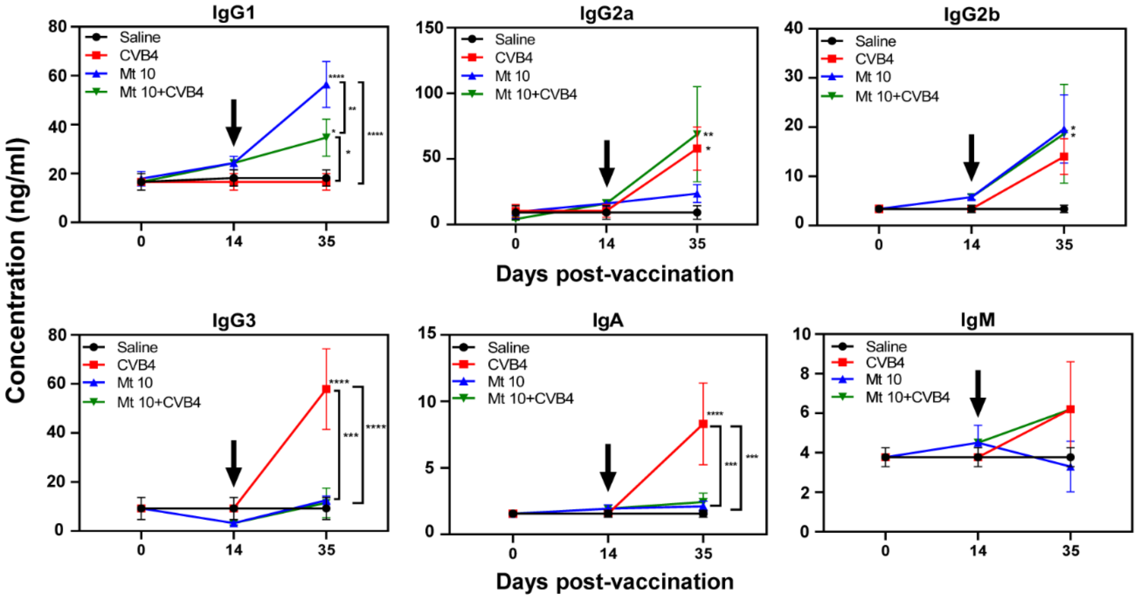

Figure S1. Detection of serum Ig isotypes. Sera collected on various days from indicated groups were analyzed to quantify Ig isotypes using LEGENDplex bead array as described in the methods section. Mean ± SEM values obtained from three individual experiments, each involving n = 3 to 6 mice are indicated. Black downward arrow indicates time-point of CVB4 challenge. Two-way ANOVA and Mann-Whitney test were used to determine significance between groups. *p ≤ 0.05, ** p ≤ 0.01, ***p ≤ 0.001 and ****p ≤ 0.0001.

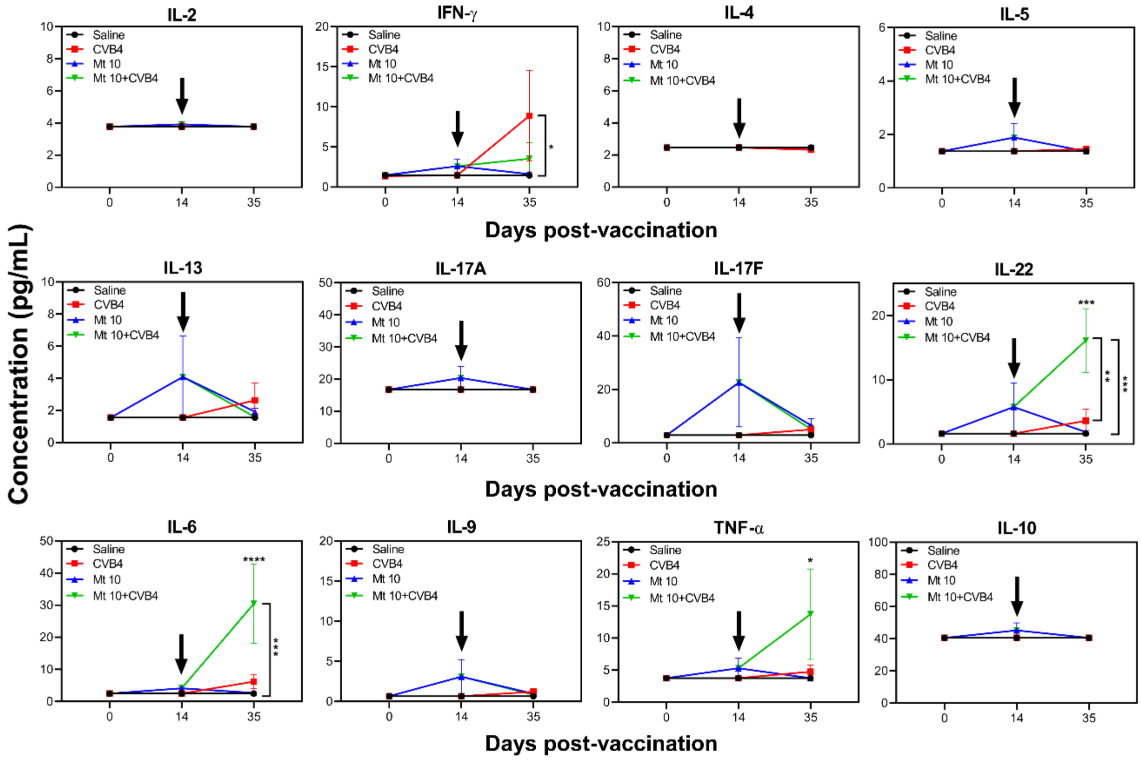

Figure S2. Serum cytokine analysis of vaccinated mice. Sera collected on days 0, 14, 35 post-vaccination from the indicated groups were analysed for cytokines using LEGENDpleax cytokine bead array as described in the methods section. Mean ± SEM values obtained from three individual experiments, each involving n = 3 to 6 mice are indicated. Black downward arrow indicates time-point of CVB4 challenge. Two-way ANOVA was used to determine significance between various treatment groups. *p ≤ 0.05, **p ≤ 0.01, ***p ≤ 0.001 and ****p ≤ 0.0001.
